# Growth of the Cementum

**Published:** 1891-09

**Authors:** R. R. Andrews

**Affiliations:** Cambridge, Mass.


### GROWTH OF THE CEMENTUM.[1]

BY R. R. ANDREWS, D.D.S., CAMBRIDGE, MASS.

In the year 1858, Magitot, a French histologist, claims to have found within the follicle of a developing tooth a special organ for the development of the cementum. In 1861, Robin and Magitot made a presentation of the same facts anew. With the exception of these authors, I am not aware that any other authority has recognized the presence of this special organ; while such men as Kölliker, Waldeyer, Herz, and others have formerly denied its existence. In my own investigations I have not been able to trace it with certainty, although there are appearances in a fully-formed follicle of a tissue under the calcifying dentine-germ, between it and the outer covering of the sacculus, that might admit of the supposition of the existence of such an organ. I have noticed this appearance in sections from embryos from the pig and the calf. At a later stage, where the crown is further developed, there is also to be seen infoldings of the tissue at the base of the germ that may develop into an organ for the growth of the cementum, as stated by Magitot. But in teeth more matured, where the cementum has already commenced its growth, I cannot trace even the outlines of a special organ, although I do not consider my investigations to have been extensive enough to warrant me in denying its existence altogether.

Wedl, whose description of the development of the dentine and the enamel is so minute, has but little to say about the development of the cementum. He tells us that at the margin of the crown the dental sacculus contracts, and upon its inner surface the

---

[1] Read before the Section of Dental and Oral Surgery of the American Medical Association, held in Washington, D. C., May, 1891.

formation of the cement is affected, increasing gradually as the formation of the root advances. The lower segment of the dental sac becomes therefore the root membrane of the tooth. He believes with Tomes that Nasmyth's membrane belongs to the cement. Again, he states that the dentine and cement are connected together by means of a layer composed of an agglomeration of transparent globules of varying degrees of thickness. The spaces intervening between the latter (interglobular spaces) are irregularly notched, and frequently in very close proximity to one another. He considers that the cement commences outside of this layer, but some of my own sections show it as a dividing line. In describing the methods in which hypertrophy of the cement is formed, he speaks of the various sizes of the corpuscles which form it, stating that many of them have a glistening appearance. Smaller corpuscles are sometimes attached to the sides of larger ones, or are blended with them. Large and small ones also occur separately. These so-called corpuscles are, I believe, globules of calcoglobulin, forming, by merging into others, a layer of calcoglobulin that shall form the matrix of the hypertrophied cement.

Tomes tells us that it is difficult to point out any distinguishing structural character between primary bone and that of the cementum. The cells close to the surface of the forming cementum which were formerly called osteal cells have now been named osteoblasts. No bone is formed until after the appearance of this osteoblast tissue, and Rollet believes that these osteoblasts are essentially a new growth; they are so distinctly marked off that it almost assumes the character of an epithelium. If we harden a partially-formed tooth in chromic acid, and subsequently decalcify and cut it transversely through the root, we meet with the following structures from without inward: On the outside is the outer part of the sacculus, now the periosteum. Internal to this is a layer to which the name cambium has been given, consisting of roundish cells with processes. These lie in a reticulum made up of cells which give out a small number of homogeneous transparent processes. By the inosculation of these processes a net-work is formed. Between this net-work and the fully-formed cementum lies the osteoblast layer, consisting of much larger cells. As the osteoblasts form a continuous layer and are very numerous, it is obvious that only a small percentage of them ever form lacunæ, or bone cells, or otherwise retain their individuality. As the process of calcification goes on, the outlines of individual cells become lost in the general transparency of the matrix, only a cell here and there remaining as a lacuna. Again, he states that contiguous osteoblasts become fused together by their exteriors, so that their individuality is lost.

Professor James Tyson, in 1873, writes: "Some difference of opinion existed as to the exact tissue which undergoes conversion into cementum, some alleging that it results from ossification of the tooth-sac, while others, among whom are Kölliker and Beale, believe it to originate in a soft stellate tissue, made up of branching and communicating cells, which are found upon the surface of roots of teeth, and within the tooth-sac. This tissue undergoes calcification, spherules of lime being deposited, which gradually fuse and form a transparent, intercellular substance. In this process not all the cells of the stellate tissue become lacunæ of the cementum, but some are obliterated by the deposit, and there are therefore fewer lacunæ in the resulting cementum than in the previous stellate tissue, while the canaliculi are much more numerous than the prolongation of the stellate cells, many of the lacunæ having thirty or forty prolongations, while the stellate cells rarely have more than from ten to twelve. The cementum is more slowly formed than bone and a more permanent, but probably less perfect, tissue; its matrix is harder and more transparent, in this respect approaching the dentine."

Klein tells us that the tissue of the tooth-sac represents the matrix from which the cement is formed; its structure and function are that of the osteogenetic layer of the periosteum, and that the formation of the cement out of that tissue is identical with subperiosteal bone.

My investigations show that, if we examine the developing tooth just after the cementum has commenced to form, we shall find that the matrix of the cementum is made up of masses or layers of that tissue which we find everywhere on the borderland of calcification, between the organic and inorganic substance. This is a tissue which Tomes has said was produced solely by the destructive action of weak acids, but that his conclusions are erroneous is proven by the fact that this tissue appears in sections where no acid has been used. It is a tissue formed by the coalescing of minute, globular bodies, calcospherites, into globules and layers of a tissue called calcoglobulin. The minute globules, in forming the matrix of cementum, seem to come from the osteoblasts, and form the calcoglobulin layer in somewhat the same manner as I have described and pictured in the developing dentine. The smaller globules by merging into each other form larger ones.

They have a glistening appearance, like fat-cells, are about the size of the osteoblasts, but are not cells, though often taken for them. They have no membrane and are without a nucleus. They are the bodies which Tomes and others say become fused together by their exteriors. Outside of the layer formed by the globules, the developing matrix of the cementum, we see a row of cells, which Rollet stated looked like an epithelium. They are the osteoblasts, or cementoblasts, and the granules that have been described in their substance are very minute calcospherites, which the cells give out to the forming matrix. Tomes has called them osteal cells. They are the same in appearance as those we see around the edge of the developing bone of the jaw. Just exterior to these cells we find roundish, nucleated cells with innumerable processes, reminding one somewhat of a stellate reticulum, only that the stellate character is not so marked. Just outside these we find the connective tissue which is really the periosteum. If there exists at this time a special cement organ, it must be formed by this slight amount of stellate tissue which is between the periosteum and the layer of osteoblasts that are against the forming cementum. In preparing my tissue, so as to have it as near life as possible, I made use of the same methods as were described in my papers on the development of the dentine and of the enamel, and I find that the osteoblasts, which are said to be full of a peculiar granular substance, are, when the tissue has been properly prepared, found to be filled with minute spherical bodies, which have a glistening appearance. Across the developing matrix of the cement are found numerous fibres, probably connective-tissue fibres, that are also found in developing bone. They were seen and described by Sharpey, and are named after him, Sharpey's fibres. They become calcified within the matrix.

As the cementum grows thicker we find that the developing matrix is infolding in its substance large nucleated bodies which appear to be connective-tissue corpuscles. They are somewhat larger than the osteoblasts, and are forming the cement cells, or lacunæ. They have a higher function than the osteoblasts,—that is, they are to give nourishment to the matrix, being connected with others by means of canals or processes, of which there are many, some of which run in the direction of the termination of the dentinal tubes, as though connected with them. When enclosed by the developing matrix of the cement, minute, glistening, globular bodies are seen within their outline, or membrane; indeed, it appears as though these minute globules were deposited on the periphery of the cell by the cell itself, and here fusing, give the cell its peculiar characteristic shape, which is not as regular as that in bone, and is oftentimes very much larger. Their processes, probably, anastomose with the dentinal tubules through the interglobular spaces of the so-called granular layer, although I have never been able to trace them. I look upon the granular layer itself as a condition caused by an arrest of the developmental process, while the first layers of the dentine were being formed. It has its existence solely from the fact that in the first-forming layer of dentine the globules or calcoglobulin which form its matrix did not fuse together. It is exactly identical to the interglobular spaces found in the crown. The point that I would emphasize in this paper is that the matrix of the cementum is formed from a secretion of the osteoblasts, and this secretion is a multitude of minute globular bodies given out against the dentine. In a work entitled "General Biology," written by Professors Sedgwick and Wilson, they make a statement that the matrix of a tissue is composed of lifeless matter, which has been manufactured and deposited by the living protoplasm, constituting the bodies of the cells; and again, cells may manufacture a lifeless substance which appears in the form of solid partition walls between the cells, or as a matrix solid or liquid in which the cells lie; and again, the cells are small masses of living matter or protoplasm, which deposit more or less lifeless matter either around (outside) them or within their substance. The lifeless matter which is given out by the osteoblasts to form the matrix of the cementum is in the form of these minute globules, and these fusing together form larger ones, which, merging, form layers of an uncalcified substance that Professor Harting has named calcoglobulin; this is by further calcification to become the calcified matrix. In speaking of the formation of exostosis, Tomes states that at the junction with the root he finds a substance that is dense. It is torn with difficulty, and under pressure slips about between the two glasses. It is gelatinous, osseous matter, and with it may be seen rounded, amorphous molecules. This is a good description of calcoglobulin. Tomes says that cementum is not developed by a direct metamorphosis of the periosteum, but by the calcification of a new growth. Cells are produced, the individuality of which becomes lost in the process of calcification; the interior of the cells seems stuffed with an opaque and dense substance disposed in large granules, among which the nuclei cannot positively be pointed out. The large granules within the cells are really calcospherites, and careful preparation of the tissue will clearly show them. The merging together of these globules forms a layer, and this calcifying, layer after layer, gives to the cement the peculiar laminated appearance that is so often seen. It is by no means difficult to trace evidences of this globular formation in the cementum of fully-formed teeth; indeed I have several sections of human teeth that show the outlines very clearly,—so clearly, in fact, that one might call it the interglobular spaces of the cement, probably caused from some arrest in the full development of the tissues.

